# Cost-Effectiveness Analysis of Trastuzumab in the Adjuvant Treatment for Early Breast Cancer

**DOI:** 10.5539/gjhs.v7n1p98

**Published:** 2014-08-15

**Authors:** Ali Aboutorabi, Mohammad Hadian, Hossein Ghaderi, Masoud Salehi, Maryam Ghiasipour

**Affiliations:** 1 Department of Health Economics, School of Health Management and Information Science, Iran University of Medical Sciences, Tehran, Iran; 2 Department of Statistic and Mathematics, School of Health, Tehran University of Medical Sciences, Tehran, Iran; 3 Department of Health Management and Economics, School of Health, Tehran University of Medical Sciences, Tehran, Iran

**Keywords:** adjuant therapy, cost-effectiveness, early breast cancer, trastuzumab

## Abstract

**Background:**

Evidence from randomized controlled trials (RCTs) has shown a significant survival advantage of trastuzumab. Although extant work in developed countries examined economic evaluation of trastuzumab in adjuvant treatment for early breast cancer based on the 1-year treatment, there is uncertainty about cost-effectiveness of trastuzumab in the Adjuvant Treatment of early breast cancer in developing countries. This study aimed to estimate cost-effectiveness of adjuvant trastuzumab therapy compared to AC-T regimen in early breast cancer in Iran.

**Methods:**

A cost-effectiveness analysis was performed using a Markov model to estimate outcomes and costs over a 20-year time period using a cohort of women with HER2 positive early breast cancer, treated with or without 12 months trastuzumab adjuvant chemotherapy. Transition probabilities were derived mainly from the BCIRG006 trial. Costs were estimated from the perspective of the Iranian health care system. Both costs and outcomes were discounted by 3%. One-way sensitivity analysis was undertaken to assess the associated uncertainties in the expected output measures.

**Results:**

On the basis of BCIRG006 trial, our model showed that adjuvant trastuzumab treatment in early breast cancer, yield 0.87 quality-adjusted life-years (QALY) compared with AC-T regimen. Adjuvant trastuzumab treatment yielded an incremental cost-effectiveness ratio (ICER) of US$ 51302 per QALY.

**Conclusion:**

By using threshold of 3 times GDP per capita, as per World Health Organization (WHO) recommendation, 12 months trastuzumab adjuvant chemotherapy is not a cost-effective therapy for patients with HER2-positive breast cancer in Iran.

## 1. Introduction

Breast cancer (BC) is the most prevalent cancer among Iranian women (Sadjadi, 2005). There were 7582 newly diagnosed cases in 2008 (Etemad, 2008-9) and a recent study by Mousavi (2009) reported an age standardized incidence rate of 28.25 per 100,000 females in 2006. Human epidermal growth factor receptor-2 is overexpressed in 25 to 30 percent of patients with primary breast cancer and it causes cancer cells to reproduce uncontrollably (D. J. Slamon et al., 1989; 2001).

Trastuzumab (Herceptin®) is a synthetic and recombinant humanized monoclonal antibody directed against the extracellular domain of the (HER2). The HER2 proteins stimulate cell proliferation and trastuzumab inhibits cell proliferation in HER2-dependent tumors (Hudis, 2007; Leyland-Jones, 2002; D. Slamon et al., 2011). Trastuzumab was approved in 1998 as a first-line treatment for HER2-positive metastatic breast cancer (Kurian et al., 2007). Then, the indication was extended to adjuvant treatment in early breast cancer.

Several randomized controlled trials (RCTs) have shown a significant survival advantage of trastuzumab, with a reduction in the rate of recurrence and improvement in the rate of survival for early breast cancer, when added to conventional chemotherapy, for 1-year(Gianni et al., 2011; Piccart-Gebhart et al., 2005; Romond et al., 2005; D. Slamon et al., 2011). As for other monoclonal antibodies, trastuzumab has a high treatment cost compared with other chemotherapeutic agents; a full course of treatment with trastuzumab is about US$70,000 (Fleck, 2006). The average wholesale price of trastuzumab is clearly above the average price of other breast cancer drugs. Therefore, better health outcomes should justify the higher treatment costs. The trade-off between the costs and benefits is a key criteria for reimbursement (Neyt, Huybrechts, Hulstaert, Vrijens, & Ramaekers, 2008). To date, there is controversy in some countries about public health funding of this drug due to its high cost and limited overall survival benefit (Fenton, 2010).

In developed countries, several studies conducted to determine economic evaluation of trastuzumab in adjuvant treatment for early breast cancer based on a 1-year treatment (Dedes et al., 2007; Garrison et al., 2007; Kurian et al., 2007; Millar & Millward, 2007; Liberato, Marchetti, & Barosi, 2007; Neyt et al., 2008; Hall et al., 2011; Skedgel, Rayson, & Younis, 2009). There is, however, uncertainty about Trastuzumab cost-effectiveness in early breast cancer for decision makers, in many developing countries (Buendía, Vallejos, & Pichón-Rivière, 2013). To the best of our knowledge, there is no study that investigated cost-effectiveness of trastuzumab treatment in Iran. To fill the gap in the literature, the current study aimed to estimate cost effectiveness of adjuvant trastuzumab therapy in early breast cancer in Iran. The findings of this study will provide useful evidence regarding efficiency of trastuzumab treatment for health care decision makers in the country.

## 2. Patient and Method

### 2.1 Model

We implemented a Markov model based on breast cancer disease states. A Markov model with four health states was designed to estimate outcomes and costs for a hypothetical cohort of women with positive early breast cancer.

**Figure 1:**
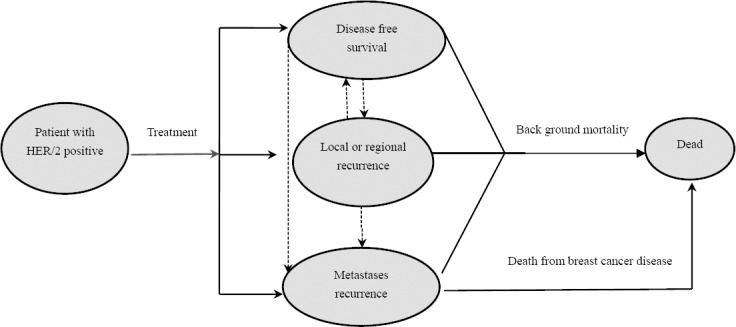
Markov model structure

As shown in Figure 1, the model included four health states: disease free state (DFS), loco-regional recurrence (local, regional and contralateral relapse), metastases recurrence and death. Based on the Markov model patients received their assigned adjuant therapy and remained in a state of disease-free state until they either died with background mortality or they experienced a loco-regional or metastatic relapse. Patients who survive with local recurrence can move to DFS or metastases state. Patients remain in a metastases state until they die from breast cancer or die from other causes. Based on BCIRG 006, congestive heart failure (CHF) was the main side-effect of both treatment taken into account.

We constructed a decision tree in TreeAge Pro 2011®, (TreeAge Software, Inc) to estimate the costs and utility effects for a hypothetical cohort of women with HER2 positive early breast cancer in two treatment strategies. According to BCIRG 006 trial, two treatment strategies were selected: adjuvant chemotherapy plus trastuzumab and adjuant chemotherapy alone. This model applied for comparing 20 years costs and effectiveness of adjuvant chemotherapy plus trastuzumab and adjuant chemotherapy alone for patients with HER2-positive early breast cancer. The average age of the population in the model was 50 years old, similar to the BCIRG 006 trials. In the first strategy, randomly assigned women with HER2-positive early-stage breast cancer received 100 mg/m2 docetaxel, 60 mg/m2 doxorubicin, 600mg/m2 cyclophosphamide intravenously (AC-T arm) in each session, six times every 3 weeks that prolonged approximately 4 months. In second strategy, the same regimen plus 52 weeks of trastuzumab (AC-T plus H) is considered. We simulated a hypothetical cohort of women with an average age of 50 year with the same entry criteria as in the BCIRG 006 trial. The incidence of adverse effects associated with the adjuvant therapy was also taken into account.

Analyses were projected to 20-year horizons. The cycle length was one year. The outcome was measured as life-year gained (LYG) and quality-adjusted life-year (QALY). Costs and QALYs were both discounted by 3%.

### 2.2 Key Assumptions

The following assumptions were made in the analysis:

- Recurrence of contralateral breast cancer was combined with local and regional recurrence.
- There were no breast cancer recurrences beyond year 20 of the model (Rivkin SE, 2003).
- The benefit of trastuzumab treatment last for 5 years (D. Slamon et al., 2011).
- Patients in the Adjuvant trastuzumab arm experience cardiotoxicity within the first year of treatment (D. Slamon et al., 2011).
- Death due to breast cancer occurs only in metastasis disease state (Millar & Millward, 2007)
- The cost of trastuzumab therapy in metastases state was omitted because less than 5% of patients in Iran use trastuzumab (Davari et al., 2013).
- Patients in local recurrence could transition to ‘distant recurrence’ while patients in the distant recurrence state remained in that state until death (D. Slamon et al., 2011)
- In BCIRG 006 study, CHF related deaths were not reported, thus mortality due to CHF was assumed to be zero (D. Slamon et al., 2011)

### 2.3 Clinical Data

The underlying clinical data and the treatment protocol, follows the BCIRG 006 trial (D. Slamon et al., 2011). Survival probabilities for disease free state in two strategies were based on a 5-year fallow-up of the BCIRG 006 trial (D. Slamon et al., 2011). Annual rate of recurrence after year 15 was obtain from Early Breast Cancer Trialists’ Collaborative Group (EBCTCG, 2005). Table 1 presents more information on the clinical inputs.

**Table 1:** Probability parameters

Parameters	Base-case estimate	Reference
Cumulative probability for DFS in AC-T group, year 5	0.75	(D. Slamon et al., 2011)
Hazard ratio for DFS in AC-TH group, year 5	0.64	(D. Slamon et al., 2011)
Hazard ratio for DFS in AC-TH group, year 6-20	1	Assumed
Annual rate of recurrence, years 5-9	4.89%	(EBCTCG, 2005)
Annual rate of recurrence, year 10-14	3.54%	(EBCTCG, 2005)
Annual rate of recurrence after year 15	2.66%	(EBCTCG, 2005)
Proportion of loco-regional versus metastases disease	0.25	(Piccart-Gebhart et al., 2005)
Incidence of congestive heart failure in AC-T group	0.7%	(D. Slamon et al., 2011)
Incidence of congestive heart failure in AC-TH group	2%	(D. Slamon et al., 2011)
Annual rate of death from metastases disease	0.328	(D. Slamon et al., 2011)
Annual rate of death from other disease	Age specific	(WHO, 2014b)

AC-T: doxorubicin and cyclophosphamide followed by docetaxel.

AC-TH: doxorubicin and cyclophosphamide followed by docetaxel plus trastuzumab.

DFS: disease free survival.

In BCIRG 006 trial at a median follow-up of 65 months, DFS rate at 5 years was 75% among the patients receiving AC-T and this rate was 84% among those receiving AC-T plus trastuzumab. Based on a same trial, the hazard ratio for recurrence in the control group, compared with the observation group, was 0.64 (95% confidence interval, 0.53–0.78; p<0.0001) (D. Slamon et al., 2011). Breast cancer recurrence rate in the first group was taken from the control group of the BCIRG 006 trial. The proportion of recurrences that are local versus metastases diseases was derived from the HERA trial (Piccart-Gebhart et al., 2005). The probability of death from metastases in both strategies obtained from the control group of trastuzumab’s RCT (D. J. Slamon et al., 2001). The proportion of patients experiencing congestive heart failure in both groups was obtained from the BCIRG 006 trial. Age specific background mortality rates for 50 to 70 year old women were derived from the Iranian life table (WHO, 2014b).

### 2.4 Quality of Life

We adjusted survival with quality of life using utility weights for years that every patient spent in each state. These weights were derived from the existent literature (see Table 2).

**Table 2:** Utility value of different health states

Health state	Base-case utility weight	Reference
Treatment With AC-T	0.94	(C. R. Earle CC, Baker CS, Bell CM, Stone PW, Sandberg EA, Neumann PJ., 2000)
Treatment With AC-TH	0.94	(C. R. Earle CC, Baker CS, Bell CM, Stone PW, Sandberg EA, Neumann PJ.)
Disease Free Survival	0.98	(C. R. Earle CC, Baker CS, Bell CM, Stone PW, Sandberg EA, Neumann PJ.)
Congestive heart Failure	0.64	(Lewis et al., 2001)
Loco- Regional Recurrence	0.615	(Lidgren, Wilking, Jönsson, & Rehnberg, 2007)
Distant Recurrence	0.615	(Lloyd, Nafees, Narewska, Dewilde, & Watkins, 2006)

### 2.5 Costs

Direct medical costs were measured from the perspective of the Iranian health care system only. Direct non-medical and indirect costs such as transportation cost, out-of-pocket payments, time spend for seeking care and loss of productivity were not measured in our study. To calculate the dosage of drugs, treatment expense for an assumed average 70kg woman was used (Larijani, 2005). Costs are expressed in US dollars. Costs were inflated to 2010 prices using the Consumer price index (CPI) for the country. Sources of cost inputs are presented in Table 3.

**Table 3:** Cost parameters

Cost description	Base case estimate (USD)	Reference
AC-T treatment overall cost	3294	(Haghighat, 2013)
Trastuzumab drug cost	48850	(ICRN, 2011)
Trastzumab administration cost	425	Local charge
Annual disease free Follow-up costs (mammography, medical visits, for 3 years)	47	Calculated based on Guideline (NICE, 2002)
Loco-regional overall cost	4138	(Haghighat, 2013)
Metastases diseases annual cost	7865	(Davari et al., 2013)
Congestive heart failure cost	675	Calculated based on Guideline (NICE, 2010)

AC-T: doxorubicin and cyclophosphamide followed by docetaxel. AC-TH: doxorubicin and cyclophosphamide followed by docetaxel plus trastuzumab.

Costs of local recurrences including costs of surgery, chemotherapy, radiotherapy, hormonal therapy and hospitalization were adopted from an earlier study in Iran (Haghighat, 2013). The costs of metastatic disease including hospitalization, surgery, chemotherapy, radiotherapy, hormonal therapy, palliative and terminal care, were derived from a cost study in Iran (Davari et al., 2013). We assumed that adjuvant hormone therapy with an aromatase inhibitor was received for 5 years by 70% of the patients in both groups (Haghighat, 2013). The cost of trastuzumab therapy was not considered in local and metastatic phases. Other local costs of Trastuzumab therapy in the adjuvant setting including HER2/neu screening, drug administration, supportive medications, and patient management, were obtained through a general hospital (Imam Khomeini hospital) in Tehran city. Costs of managing congestive heart failure were calculated based on international guideline (NICE, 2010).

The costs of follow-up examinations for disease-free patients were calculated according to international guidelines and based on local charge (NICE, 2002).

### 2.6 Sensitivity Analysis

To assess the robustness of the study results, one-way sensitivity analyses were performed. We changed input parameters between the upper and lower frontier according to experts’ advice, and explored the results.

## 3. Results

The model results was expressed in terms of life years saved (LYs), quality-adjusted life years (QALYs) and incremental cost-effectiveness ratios (ICER). The results of the analysis are summarized in Table 4.

**Table 4:** Base case results

Parameters	AC-T	AC-TH	Difference	ICER (USD)
20-year treatment and fallow-up costs (USD)	12388	56984	44596	---
Life years gained (LYGs)	11.81	12.63	0.82	54223
Quality-adjusted life years gained (QALYs)	11.11	11.98	0.87	51302

In the base case analysis (i.e. over 20-year time horizon) the QALYs gained with AC-T regimen (strategy 1) and with 52 weeks adjuant trastuzumab treatment for HER2-positive patients (strategy 2) were 11.11 and 11.98, respectively. Therefore, the new intervention produced an extra 0.87 QALYs. The total costs for AC-T adjuant and AC-T adjuant treatments were 12,388 USD and 56,984 USD, respectively. The base case analysis indicated that treatment with a 12-month adjuvant trastuzumab regimen generated an ICER of 51,302 USD per QALY.

### 3.1 Sensitivity Analysis

One-way sensitivity analyses were undertaken to assess robustness of the study results. Result of this analysis presented in Table 5.

**Table 5:** Results of sensitivity analyses in incremental cost-effectiveness ratios (ICER) (USD)

Variable	Lower	Upper
Cost of trestuzumab(-30%/+30%)	34296	68307
Cost of treating metastases(-30%/+30%)	50807	51795
Discount rate (0%/ 6%)	37775	67661
Cost of congestive heart failure(-30%/+30%)	51159	51403
Utility Weight of DFS (0.75/1)	50174	69169
Utility Weight of metastases (0.45/0.75)	49588	52794
Hazard ratio for DFS (0.37/.64)	43887	51302

The ICER for the base case = 51302 USD/QALY.

Sensitivity analyses of variables in the model showed that model is sensitive to changes in the cost of trastuzumab, discount rate for outcomes and hazard ratio in AC-TH group. In order to assess model validation, the results of the model for two treatment strategies were compared with overall survival at 5 years in BCRG006 trial. Overall survival at 5 years in BCRG006 trial reported 92% and 87% in AC-T and AC-TH group. In our Markov model, overall survival in same period is 93% and 85%.

## 4. Discussion

In developed countries, 1-year adjuvant trastuzumab treatment is cost-effective in early breast cancer treatment, particularly in a long-term perspective (Garrison et al., 2007; Hedden et al., 2012; Liberato et al., 2007). This costly adjuvant treatment in early breast cancer reduces risk of metastasis disease and improves overall survival (D. Slamon et al., 2011). In developing countries, however, there is some uncertainty about the cost–effectiveness of this regimen (Buendía et al., 2013).

In base case analysis, our model yielded an ICER with 51302 USD per QALY after 20 years for the BCIRG-006 trial. High cost–effectiveness ratio yielded in our study is comparable with similar studies (Garrison et al., 2007; Hedden et al., 2012; Liberato et al., 2007) evaluating cost-effectiveness of trastuzumab conducted in developed countries. For example, a study in Italy by Liberato and colleagues (Liberato et al., 2007) with 5-year benefit duration and with 15-year time horizon, cost per QALY estimated 22,385 USD. They concluded that adjuvant trastuzumab treatment in early breast cancer is cost-effective. This estimate is lower than our ICER estimate. The difference between this finding and our result can be explained by the shorter time horizon and lower relative risk applied in trastuzumab group in the study by Liberato et al.(2007). Moreover, according to HERA trial, relative risk of any relapse was 0.4 in the Liberato et al. study. Additionally, in the Italian study, high cost of trstuzumab was estimated in metastatic disease treatment.

In a study by Garrison et al. (2007), with a 20-year time horizon, the incremental cost per QALY was calculated 34,201 USD. Some of the assumptions used in the Garrison et al. study is similar to our study, such as time horizon (20 years), length of benefit duration (5 years) and discount rate (3%). However, their study is constructed upon the joined analysis of the NCCTG N9831 and NSABP trials. Therefore, transitional probability for disease free survival in their study differs from the input we used in our analysis.

In a study by Hedden and colleagues (Hedden et al., 2012), with 5-year benefit duration and 5% discount rate and 28-year time horizon, the cost effectiveness of trastuzumab was calculated 13,095 USD per QALY.. Lower cost-effectiveness ratio in this study, comparing with other similar studies, is due to high survival rate of patients and cost of trastuzumab for relapse treatment (Hedden et al., 2012). In Iran, less than 5% of patients, particularly in relapse state, receive trastuzumab (Davari et al., 2013). Majority of patients receive a cheaper alternative treatment regimen, such as standard chemotherapy plus taxane. Therefore, we did not calculate cost of trastuzumab in the metastases state. We believe that the main reason for the high cost-effectiveness ratio in our analysis, compared to the similar studies in other settings, is lower treatment costs for metastases state. Some part of the lower costs, compared with developed countries settings, can be explained by the subsidized health care system and also lower cost of services in Iran. For example, in Dedes et al.(Dedes et al., 2007) study, costs of treating metastatic estimated 41,412 EUR, while these costs was estimated less than 20% of this figure in Iran.

Majority of economic evaluations studies in developed countries have predicted that adding trastuzumab to chemotherapy is cost-effective relative to other breast-cancer therapies. Trastuzumab remained a cost-effective treatment strategy in developed countries with a high willingness-to-pay threshold. For example, the National Institute for Health and Clinical Excellence (NICE) estimated that adjuvant trastuzumab treatment in the United Kingdom has an incremental cost per QALY gained of £18,000, which is less than the general £30,000 threshold (NICE, 2006). Methodologies and the geographic contexts conclusions may differ between developing countries reports as a result of different assumptions. In a study in Colombia, based on therapy regimens and results from the 4 year fallow up of the NCCTG N9831 and NSABP trials, Buendia and colleagues estimates that adjuvant trastuzumab has an incremental cost per QALY gained of USD71491. Buendia and colleagues (2013) used a 20-year horizon in the base case, 5% discounted rate, and also assumed no additional trastuzumab benefit after 5 years. The QALY differential estimate from our study is similar to Buendia et al’s study. Buendia et al’s projected differential in discounted QALYs was 0.80 versus 0.87 in the present study. Our base case estimate for the ICER is USD 51302, which is lower than the estimate calculated in the Colombian study, due in part to the different cost estimation, different discount rate and different hazard ratio. Buendia and colleagues concluded that trasutuzumab is not a cost-effective therapy for HER2-positive, early breast cancer-adjuvant.

Willingness-to-pay and acceptable threshold in developing countries such as Iran and Colombia has not been determined yet. Therefore, we used the WHO cost-effectiveness threshold (i.e., less than 3 times GDP per capita of the country) for relative cost-effectiveness in our study (WHO, 2014a). This threshold in Iran would be lower than developed countries. According to experts’ opinion this threshold for Iran estimated to be between 10,000 to15,000 USD (WHO, 2014a).

Since 2007, the Iranian Ministry of Health and Medical Education provides some financial supports for patients but trasutuzumab is not covered with current insurances schemes. The high price of this drug can impose catastrophic expenditure to the patients and their households.

One of the strengths of our study was that our clinical data were derived from 5-year analyses of BCIRG006 clinical trials, while most of the cost-effectiveness analyses done on trastuzumab, are based on a one-year HERA trials. Our analysis, nevertheless, has few limitations need to be acknowledged. First, we estimated only the direct medical costs from the health care system perspective and direct non-medical and indirect costs such as transportation cost, out-of-pocket payments, time spend for seeking care and loss of productivity were not measured in our study. Indirect costs and productivity gains are possibly substantial and adding these costs to the model would have increased lifetime costs. Second, some of the cost components such as the costs of managing congestive heart failure and cost of follow up for disease-free patients, were estimated based on international guidelines and may not reflect the real costs. Finally, due to the lack of utility weights for the breast cancer patients in Iran, utility weights were obtained from other settings than Iran. Geographical and cultural difference between countries may affect utility weights.

## 5. Conclusion

Using WHO cost-effectiveness threshold base on GDP per capita, 12 months trastuzumab adjuvant chemotherapy in early breast cancer is not a cost-effective therapy for the patients with HER2-positive in Iran.

### 5.1 Ethical Issues

This study was approved by the Ethics Committee of Iran University of Medical Sciences.
